# Congenital syngnathia: a case report of osseous fusion between the maxilla and mandible

**DOI:** 10.3389/fped.2026.1787330

**Published:** 2026-03-30

**Authors:** Haoran Wang, Li Zhang, Jingtao Li

**Affiliations:** 1 Department of Radiology, West China Second University Hospital, Sichuan University, Chengdu, Sichuan, China; 2 Key Laboratory of Birth Defects and Related Diseases of Women and Children (Sichuan University), Ministry of Education, Chengdu, Sichuan, China; 3 Department of Neonatology, West China Second University Hospital, Sichuan University, Chengdu, Sichuan, China; 4 State Key Laboratory of Oral Diseases & National Center for Stomatology & National Clinical Research Center for Oral Diseases & Department of Oral and Maxillofacial Surgery, West China Hospital of Stomatology, Sichuan University, Chengdu, Sichuan, China

**Keywords:** case report, craniomaxillofacial surgery, mandibular reconstruction, pediatrics, radiology

## Abstract

Congenital fusion of the maxilla and mandible is an extremely rare craniofacial anomaly that can present at birth with trismus and feeding difficulties. We report a case of a neonate with right-sided osseous fusion between the maxilla and mandibular body, initially unrecognized at multiple centers. The patient developed severe hyperbilirubinemia and dehydration due to impaired feeding. Computed tomography with three-dimensional reconstruction confirmed the diagnosis and revealed no involvement of the temporomandibular joint or tooth buds. Ultrasonic osteotomy was used to resect the fusion, restoring partial mouth opening and enabling enteral feeding. This case emphasizes the importance of early imaging for accurate diagnosis and timely multidisciplinary intervention. Prompt recognition and surgical correction can improve oral function and help prevent potential neurologic complications, highlighting a critical diagnostic and therapeutic pathway for this rare but manageable condition.

## Introduction

Congenital syngnathia is a rare craniofacial developmental anomaly characterized by abnormal fusion between the maxilla and the mandible; it can present as either fibrous adhesions or bony fusion (1). Bony fusion is even rarer and can severely impair mouth opening, breathing, and feeding in affected infants. Prompt surgical intervention is required in severe cases (2). Imaging plays a crucial role in determining the type and extent of fusion, as well as in preoperative planning.

We report a full-term newborn with congenital bony syngnathia who presented with restricted mouth opening and jaundice. The diagnosis was confirmed by orofacial computed tomography (CT) scanning.

## Case description

**Figure 1:**
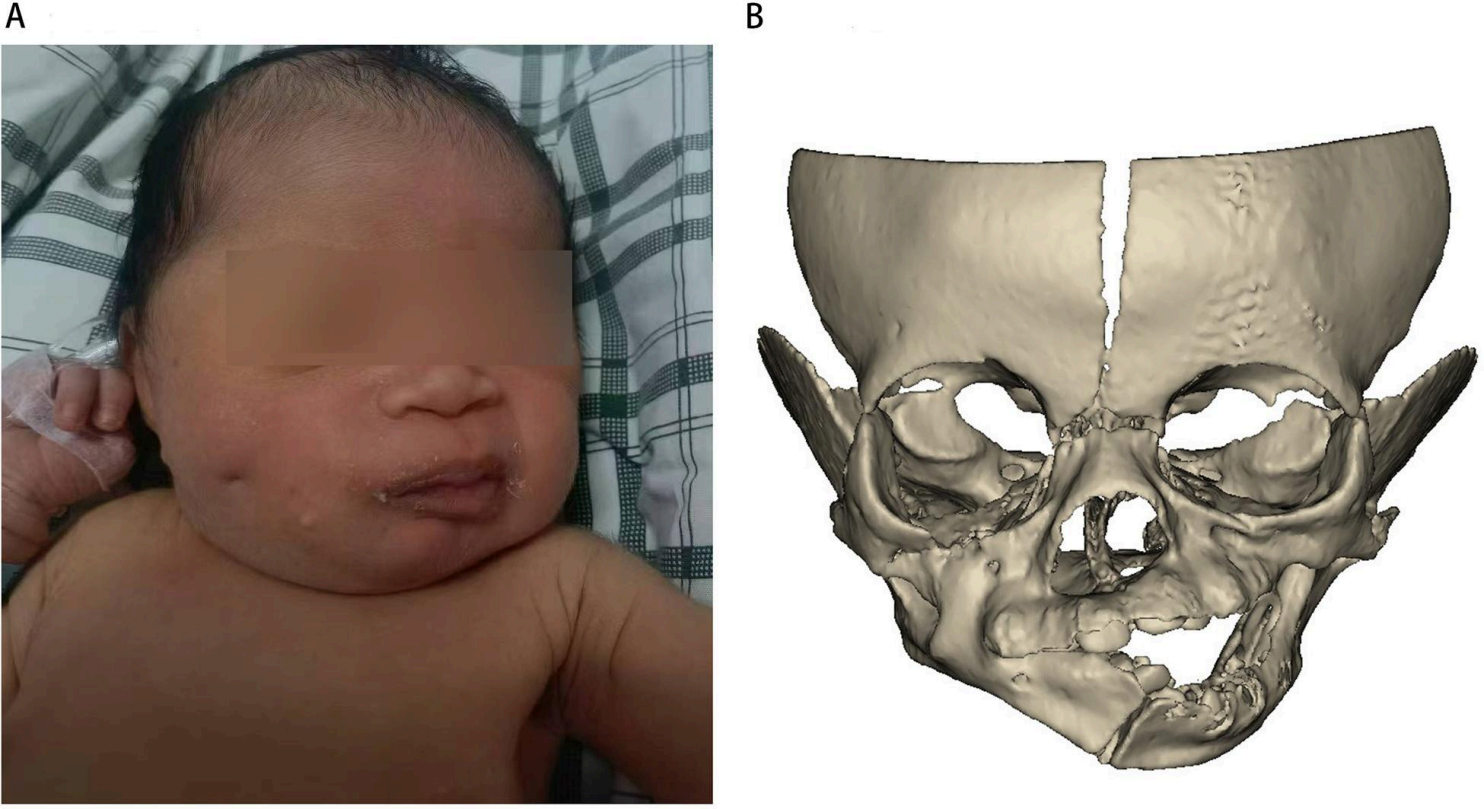
**(A)** Frontal photograph of the neonate showing limited mouth opening and facial jaundice. **(B)** Frontal 3D CT reconstruction demonstrating unilateral bony fusion on the right side.

A 9-day-old female neonate was admitted to our NICU because of “difficulty in opening the mouth for 9 days and jaundice for 8 days after birth.” The baby was born at 39 6/7 weeks' gestation with a birth weight of 3,500 g. Her Apgar scores were 9 at 1 min and 10 at 5 min. The physical examination at admission revealed a weight of 2,890 g, fair reactivity, heavily jaundiced skin with poor elasticity, limited mouth opening (Figure 1A), adhesive upper and lower gums, and oral secretions. Two fistulas were seen on the right cheek. The head circumference was approximately 33 cm, which is within the normal range for a term neonate. Routine newborn hearing screening using auditory brainstem response (ABR) yielded normal results. The cardiopulmonary examination revealed no abnormalities. The family history, psychosocial background, and perinatal course were unremarkable. Total bilirubin was measured at 420.1 µmol/L in laboratory tests, with indirect bilirubin levels of 393.0 µmol/L, and a Na^+^ level of 155 mmol/L. Prior to admission to our institution, the infant had been evaluated at multiple primary care and regional hospitals. However, the rare nature of the maxillofacial malformation went unrecognized and untreated, and oral feeding was never successfully established. As a result, the infant's jaundice and dehydration progressively worsened. The rarity of this anomaly and the subtlety of its early signs contributed to delayed recognition at multiple institutions prior to admission (1).

**Figure 2:**
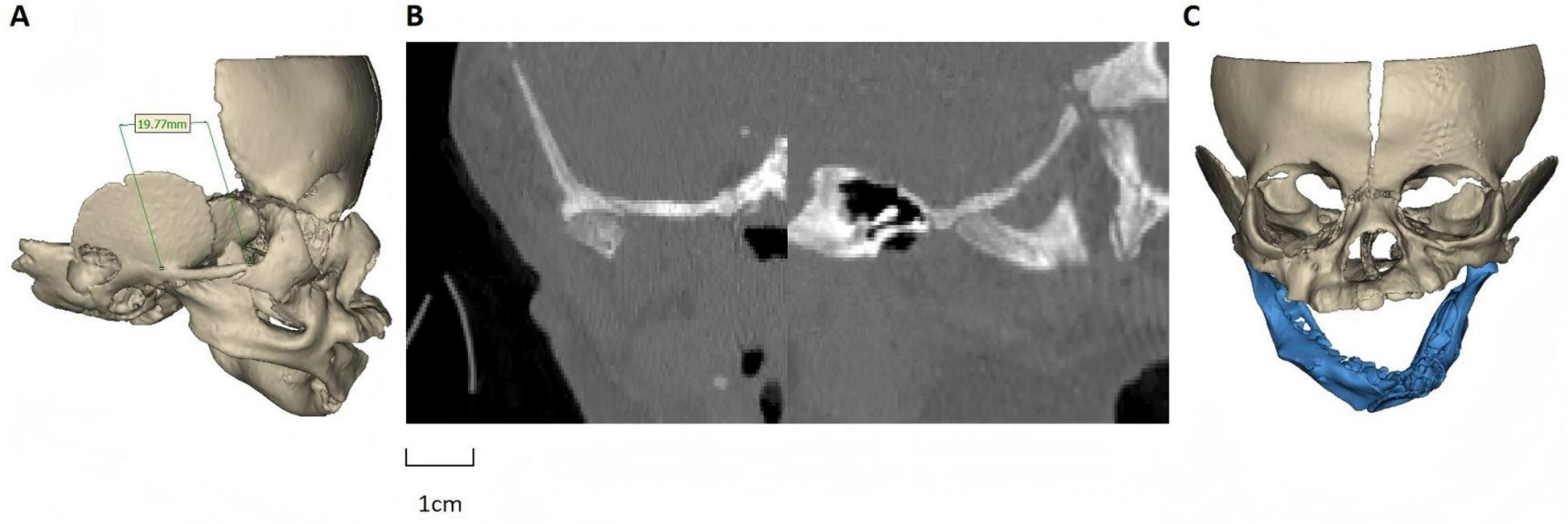
**(A)** 3D CT image of the right side showing the osseous bridge between the maxilla and mandible. **(B)** Sagittal CT image demonstrating the bony fusion with a normal temporomandibular joint (scale bar = 1 cm). **(C)** Simulated mouth opening on 3D CT after virtual osteotomy.

Orofacial CT scanning and three-dimensional CT demonstrated a 10-mm-wide continuous bony fusion between the right maxilla and the mandibular body (Figures 1B, 2A). The bony fusion did not contain any tooth buds. The mandible displayed asymmetry, characterized by leftward tilting of the occlusal plane. An open bite was observed on the left side. No signs of ankylosis were detected in the temporomandibular joint region (Figure 2B). The mandible was freed and rotated to mimic mouth opening (Figure 2C), and the interference between the coronoid process and the zygomatic body was excluded. Three-dimensional imaging is highly advantageous in assessing the range of osseous adhesion (3), thus effectively assisting in preoperative planning and intraoperative navigation.

**Figure 3:**
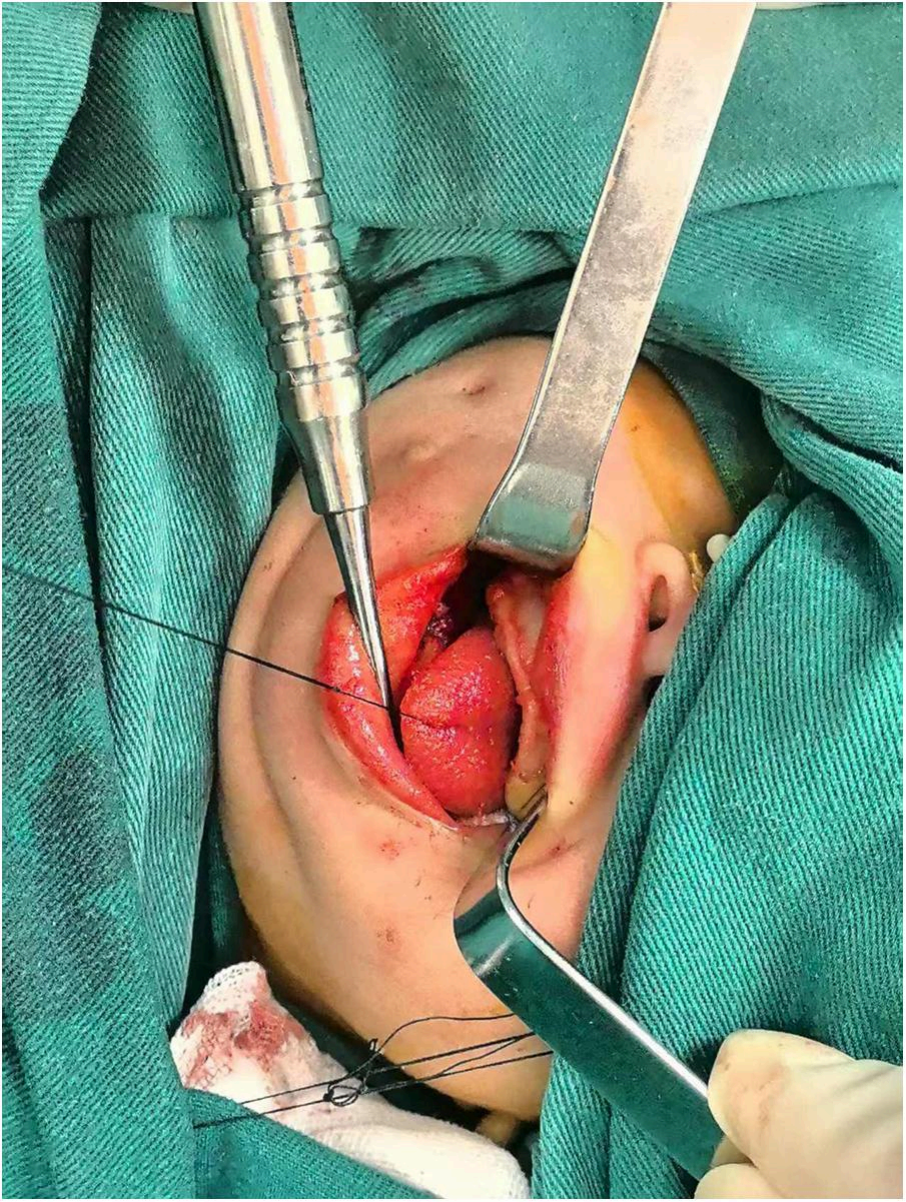
Intraoperative view showing improved mouth opening after resection of the osseous bridge.

Diagnosis: (1) congenital bony fusion of the maxilla and the mandible; (2) neonatal severe hyperbilirubinemia; and (3) moderate-to-severe hyperosmotic dehydration. The maxillofacial surgeons resected the intermaxillary bony fusion via intraoral access with a piezosurgical device under general anesthesia. A mouth opening of 10 mm was achieved on the surgical table (Figure 3).

“Right maxillomandibular osteotomy + right fistula and the left accessory auricle resection” was performed on the patient under general anesthesia. During the surgery, the bone bridge at the fusion of the maxilla and the mandible was exposed through an intraoral incision. The bone bridge was precisely excised using an ultrasonic osteotome. This method was chosen to maintain the integrity of normal bone margins and preserve jaw contour. Part of the wound was covered with a fascia flap and repaired with a tissue patch. The patient received passive mouth-opening training and swallowing function training after the surgery, and progressively transitioned to oral feeding. She was discharged after 29 days with a body weight of 4,100 g, corresponding to an average daily weight gain of 42 g. At the last follow-up, the infant was 6 months old and breastfed exclusively with normal growth and development. The patient adhered well to postoperative rehabilitation, and training was completed as instructed with no adverse events observed, and no concerns regarding hearing or early speech perception reported by the parents. The timeline of clinical events is shown in Table 1.

**Table 1:** Timeline of clinical events.

Time point	Clinical event
Birth	Born at term, weighing 3,500 g, with an Apgar score of 9/10
Day −9	Difficulty in opening the mouth
Day −8	The skin appears yellowish
Day 0	The child patient was admitted to the hospital with a weight of 2,890 g, showing a good response, jaundice, and dehydration; CT scan indicated bony fusion of the upper and lower jaw bones
Day +1	Initiate supportive treatments such as phototherapy, anti-infection therapy, and intravenous nutrition
Day +4	Perform three-dimensional CT to confirm the fusion position and the morphology of the bony bridge, and exclude involvement of the temporomandibular joint
Day +7 (Procedure day)	“Right maxillomandibular osteotomy + right fistula and the left accessory auricle resection” was performed on the patient under general anesthesia.During the operation,the bony bridge was completely removed
Day +13∼28	The patient recovered well with normal body temperature and stable breathing, and could be fed orally
Day +29	The patient was discharged with a weight of 4,100 g

## Discussion

Congenital fusion between the maxilla and mandible represents an uncommon craniofacial anomaly. It can present as either a fibrous adhesion or a bony fusion, with the latter being particularly uncommon. This condition is most commonly bilateral and often associated with syndromes related to abnormalities of the first and second branchial arches (1, 2). The present case, featuring a unilateral bony fusion on the right side combined with a contralateral buccal appendage and a right buccal fistula, suggests a complex developmental anomaly potentially involving multiple branchial arches, which is an extremely rare presentation in existing reports (2, 4).

Imaging plays a vital role in both diagnosis and preoperative assessment of this condition. Three-dimensional CT reconstruction continues to serve as the gold standard for detecting osseous fusion, as it allows precise evaluation of the location, extent, and morphology of the bony bridge, as well as its relationship with adjacent anatomical structures, thereby informing individualized surgical planning (1).

The bony bridge was surgically excised using an ultrasonic osteotome technique. Following excision, the wound was covered with a fascial flap and reconstructed using tissue patches to maintain facial anatomy and facilitate postoperative healing. Post-surgical oral mobility exercises and swallowing therapy enabled the patient to achieve significant enhancement in mouth opening capacity, successfully progress to a full oral diet, and attain appropriate weight gain. These findings suggest that early surgical intervention for such conditions can produce positive outcomes.

Although congenital maxillomandibular fusion is rare, existing findings suggest that most affected infants can achieve satisfactory outcomes in terms of mouth opening and facial development when early surgical intervention is combined with standardized rehabilitation therapy (3, 5). However, due to the rarity of the condition and the subtlety of early signs, misdiagnosis or underrecognition at initial presentation remains a significant clinical issue (6). In this case, the patient had been evaluated at multiple healthcare institutions prior to admission, but the structural abnormality was not identified, and she remained unable to feed orally. As a result, the infant developed severe hyperbilirubinemia and dehydration, placing her at potential risk for kernicterus and irreversible neurologic injury (7, 8).

Therefore, early recognition by neonatologists and radiologists is crucial. Clinicians should remain alert to clinical signs such as restricted mouth opening and facial anomalies at birth, and promptly recommend a CT with three-dimensional reconstruction. Timely imaging can facilitate multidisciplinary intervention (9), promote early medical intervention, and strengthen family confidence. Importantly, this approach minimizes the risk of serious complications, including dehydration or metabolic imbalances resulting from feeding challenges or treatment delays.

From a clinical standpoint, congenital limitation of mouth opening in neonates requires careful differential diagnosis (10, 11). Conditions that may mimic maxillomandibular syngnathia include temporomandibular joint ankylosis, intra-oral soft-tissue synechiae and bands, severe micrognathia with glossoptosis, and syndromic craniofacial malformations (12–14). In temporomandibular joint ankylosis, the fusion typically involves the condyle and glenoid fossa, whereas in our patient, three-dimensional CT clearly demonstrated an osseous bridge between the maxillary alveolar process and the mandibular ramus with a normal temporomandibular joint (10, 11). The absence of isolated soft-tissue adhesions or clefts further supported a diagnosis of unilateral bony syngnathia rather than soft-tissue synechiae or a more complex craniofacial clefting disorder (12–14).

When compared with previously reported cases, our patient shares common features of congenital syngnathia, including early presentation with feeding difficulty, radiologic confirmation of bony fusion, and the need for timely surgical release (10, 15, 16). However, many published cases describe additional malformations such as cleft palate, limb anomalies, or defined craniofacial syndromes (15, 16), whereas our patient showed normal limb development, normal head growth and age-appropriate neurodevelopment on follow-up, consistent with an isolated form of maxillomandibular fusion. To date, no specific Online Mendelian Inheritance in Man (OMIM) entry or causal gene has been established for isolated syngnathia, although jaw fusion has been reported in several syndromic contexts (10, 12). From a genetic standpoint, congenital syngnathia has been reported both as an isolated anomaly and as part of broader craniofacial syndromes. In our patient, the absence of cleft palate, limb malformations or other systemic anomalies, together with normal head growth and neurodevelopment on follow-up support an isolated form rather than a syndromic diagnosis (12, 15). In this case, genetic testing was recommended but declined by the parents, which we acknowledge as a limitation.

The fusion pattern, anatomical complexity, preoperative imaging evaluation, and operative technique demonstrated in this case serve as an illustrative model. The early identification and treatment implemented during the neonatal period particularly contribute valuable clinical experience and imaging-based guidance for addressing comparable conditions. This report is limited by the short follow-up duration and lack of genetic testing or syndromic evaluation.

## Patient perspective

The Patient Perspective section is dictated by the patient's parents:

As the parents of the patient, we were initially horrified and perplexed upon seeing her facial deformities, inability to open her mouth for normal feeding, and even symptoms such as jaundice. After her diagnosis and successful surgery, we were deeply relieved to see her symptoms significantly improve, able to eat normally, and grow like other babies. We are extremely grateful to the medical team for saving her life.

## Conclusion

This case report describes the case of a neonate with immediate postnatal trismus and a bony fusion between the right maxilla and the mandibular body. Ultrasonic osteotomy was used to resect the fusion, and partial mouth opening was restored. This case demonstrates the importance of advanced imaging techniques for identifying and developing treatment strategies for congenital syngnathia.

## Data Availability

The original contributions presented in the study are included in the article/Supplementary Material, further inquiries can be directed to the corresponding authors.
